# Cognitive reserve in multiple sclerosis: The role of depression and fatigue

**DOI:** 10.1177/13524585251338757

**Published:** 2025-06-11

**Authors:** Clara Stein, Fiadhnait O’Keeffe, Méadhbh Brosnan, Claire Flynn, Christopher McGuigan, Jessica Bramham

**Affiliations:** University College Dublin, Dublin, Ireland; University College Dublin, Dublin, Ireland; University College Cork, Cork, Ireland; University College Dublin, Dublin, Ireland; Turner Institute for Brain and Mental Health, Monash University, Melbourne, VIC, Australia; University College Dublin, Dublin, Ireland; University College Dublin, Dublin, Ireland; St. Vincent’s University Hospital, Dublin, Ireland; UCD School of Psychology, University College Dublin, Dublin, Ireland; St. Vincent’s University Hospital, Dublin, Ireland

**Keywords:** Cognitive reserve, multiple sclerosis, depression, fatigue, leisure activities, symptom burden

## Abstract

**Background:**

Several reports suggest that cognitive reserve (CR) may protect against cognitive impairment in MS. Fatigue and depression are common in MS. Yet, their influence on engagement with activities that build CR is unclear.

**Objectives:**

This study aimed to achieve a better understanding of CR-building in MS, by examining how CR differs in people with MS (pwMS) compared with neurologically healthy individuals and by investigating how common MS symptoms interact with CR-building.

**Methods:**

In total, 206 pwMS and 150 age- and gender-matched controls participated in this cross-sectional study. Participants completed self-report measures of CR accumulated in early life and across the lifespan (including education, occupation, cognitively enriching leisure activities), and of cognitive functioning, fatigue, depression, anxiety and MS-impact on everyday life.

**Results:**

PwMS’ recent engagement in cognitively enriching leisure activities was negatively associated with self-reported cognitive difficulties (rho = −0.31, *p* < 0.001). However, after controlling for fatigue and depression, this association was no longer present. Correspondingly, we observed that higher levels of depression were associated with lower engagement in cognitively enriching leisure activities (*B* = −0.41 (95% confidence interval (CI): −0.61 to −0.22), *p* < 0.001).

**Conclusion:**

Our results highlight the importance of addressing depression and fatigue in the context of lifestyle recommendations.

## Introduction

Cognitive reserve (CR) refers to an individual’s ability to adapt cognitive processes in response to brain atrophy.^1^ This concept of CR was originally developed in the cognitive ageing literature.^2^ CR has been studied across various populations and conditions, including MS. Meta-analyses suggest that CR may help to explain individual differences in cognitive outcomes in MS, showing that higher levels of CR can protect against cognitive impairment, at least in some domains.^3,4^

An individual’s level of CR is typically estimated through proxy measures, including premorbid intellectual abilities, educational level, occupational attainment and engagement in cognitively enriching leisure activities.^2^ Traditional CR measures were originally developed for an older adult population, who potentially could have experienced a lifetime of enriching activities.

In contrast, MS is typically diagnosed between the ages of 20 and 40 years, thereby affording a different timeline for building CR.^5^ People with MS (pwMS) often experience a range of symptoms including fatigue, depression, anxiety, which impact their day-to-day functioning.^6–8^ For example, increased fatigue may be associated with lower levels of physical activity.^9^ In contrast, greater perceived self-efficacy in the management of MS symptoms can be associated with greater engagement in social and intellectual activities.^10^ As such, living with MS may impact engagement with enriching environments (CR-building) as measured through traditional CR measures.

A recent systematic review revealed that approximately half of the research on CR in MS investigated the protective outcomes of engagement in enriching activities *prior to* the MS-diagnosis (e.g. pre-MS education or leisure activities).^11^ Of the studies examining lifespan enrichment (including post-diagnosis occupation or leisure activities), only 53% accounted for the potential impact of common MS symptoms in their analyses. A minority of studies conducted analyses with and without accounting for MS symptoms. These studies highlighted that by not accounting for MS symptoms, findings about the protective nature of CR may be distorted.^12^

The present study aimed to achieve a nuanced understanding of CR-building in MS. Our main research questions addressed whether pwMS and an age- and gender-matched control group differ in CR-building, and whether the experience of common MS symptoms is associated with CR-building in pwMS. We hypothesised that pwMS and controls would not differ in early life (i.e. pre-MS) CR-building, but that pwMS would have lower levels of CR as assessed through post-diagnosis CR measures. We hypothesised that MS-impact on everyday life, fatigue, depression and anxiety would be associated with post-diagnosis CR-building in pwMS.

## Materials and methods

### Study design and participants

This cross-sectional study was preregistered (https://doi.org/10.17605/OSF.IO/J53CQ). Data were collected via an online survey hosted on Qualtrics (https://www.qualtrics.com). Data collection occurred between November 2023 and April 2024.

Participants were recruited through two streams: (1) social media, with the support of a national MS society, and (2) Prolific (https://www.prolific.com). Participation via the first stream was unpaid. Participation via the research platform Prolific was paid (£8.35/hour) as per platform standards.

PwMS and a control group of people without MS were recruited. MS diagnoses were based on self-report. Participants had to be aged 18–60 years and speak English. Participants were excluded if they reported a neurological condition likely to impact responses (e.g. stroke). Participants with neurodevelopmental conditions (e.g. attention-deficit/hyperactivity disorder (ADHD), dyslexia) were included, and this was considered in the analysis. PwMS with epilepsy were included as this could be secondary to MS, but controls with epilepsy were excluded.

The Human Research Ethics Committee at University College Dublin granted approval on 22 November 2023 (HS-23-59-Stein-Bramham). All participants gave informed consent prior to participation. A person with MS piloted our survey to ensure its acceptability.

### Measures

Demographic data were collected (age, ethnicity, gender, work status, neurodevelopmental/neurological condition, MS duration, subtype, disease-modifying treatment (DMT), last relapse, history of depression, anxiety, fatigue). All measures were completed by pwMS and controls, except for the MS Impact Scale (MSIS-29), which only pwMS completed.

#### CR measures

The Cognitive Leisure Scale (CLS) was developed for pwMS and contains seven items (e.g. reading magazines or newspapers) measuring engagement in cognitively enriching leisure activities during an individual’s early 20s (CLS 20s).^13^ Participants indicated their engagement on a 5-point Likert-type scale ranging from 1 (once/less per year) to 5 (daily) (total raw scores: 7–35). Participants also completed the CLS with reference to the last year (CLS Recent) to measure recent activity.

The Cognitive Reserve Index questionnaire (CRIq) was developed for an older adult population and assesses engagement in enriching activities across the lifespan, including years of education, work and leisure.^14^ Work activity is quantified as years spent across five levels of employment from low skilled manual to highly responsible or intellectual occupation. Leisure items assess how frequently (never/rarely vs often/always) and for how many years participants engage in weekly (e.g. domestic chores), monthly (e.g. voluntary work), annual (e.g. attending exhibitions, concerts, conferences) and fixed frequency (e.g. pet care) activities. The template used to compute standardised and age-adjusted component (CRI-Education, CRI-Work, CRI-Leisure) and total CRI scores is freely accessible (https://www.cognitivereserveindex.org). CRI scores can be categorised as low (<70), medium-low (70–84), medium (85–114), medium-high (115–130) and high (>130). Supplementary Materials 1 contains further information on CRIq administration and scoring.

#### Self-report measures

The MS Impact Scale (MSIS-29) includes 20 items on the physical and 9 items on the psychological impact of MS over the past 2 weeks.^15^ PwMS rated each item on a 5-point Likert-type scale (total raw scores: 29–145). The MSIS-29 has high internal consistency and test-re-test reliability.^15^

The Modified Fatigue Impact Scale (MFIS) consists of 21 items, constituting three subscales on physical, cognitive and psychosocial fatigue over the last 4 weeks.^16^ Participants indicated on a 5-point Likert-type scale how impacted they were by each item (total raw scores: 0–84).

The Hospital Anxiety and Depression Scale (HADS) contains two subscales screening for anxiety (HADS-A) and depression (HADS-D) symptoms experienced during the last week.^17^ Each subscale contains 7 items, rated on a 4-point Likert-type scale. Raw scores range from 0 to 21 and are categorised as subclinical (0–7), mild (8–10), moderate (11–14) and severe (15–21).^18^ The cut-off of >7 has high sensitivity (depression: 90%; anxiety: 88.5%) and specificity (depression: 87.3%; anxiety: 80.7%) in pwMS.^19^ The HADS has acceptable internal consistency.^18^

The MS Neuropsychological Questionnaire (MSNQ) is a self-report measure of cognitive difficulty, assessing how each of 15 items impacted on daily functioning over the last 3 months on a 5-point Likert-type scale.^20^ Raw scores range from 0 to 60, with a cut-off of >27 having high sensitivity (83%) and specificity (97%) for detecting cognitive difficulty.^20^ The MSNQ has high internal consistency and test–retest correlations.^21^

### Statistical analysis

Statistical analyses were conducted in R (4.4.1) using RStudio and JASP (0.19). Missing data were imputed with the median of the five nearest neighbours, identified using an extended version of Gower distance (Supplementary Materials 2 contains frequency of missing data).^22^ Missing data for pwMS and controls were imputed separately. Results are based on imputed data (Supplementary Materials 2 contains examples based on complete cases).

If test assumptions were not met, non-parametric alternatives were used.

Mann–Whitney *U* and chi-square tests examined if groups (pwMS vs controls) were age- and gender-matched. A robust 2 × 2 analysis of variance (ANOVA) (group × recruitment stream) tested if there was a main effect of recruitment stream.

Spearman’s rank correlations investigated the relationship between recent CR-building and neuropsychological outcomes in pwMS. Mann–Whitney *U* tests examined between-group differences in CR-building. Paired Wilcoxon tests examined within-group differences in early 20s (CLS 20s) versus last year (CLS Recent) engagement in cognitively enriching leisure activities.

Regression analyses (controlling for age, MS duration, time since last relapse, presence of a neurodevelopmental condition) investigated the relationship between MS symptoms and post-diagnosis/lifespan CR-building. Four multiple regression models were specified:

1. CLS Recent = *b_0_* + *b_1_*age + *b_2_*MS duration + *b_3_*time since last relapse + *b_4_*presence of a neurodevelopmental condition + *b_5_*MSIS-29 + *b_6_*MFIS total + *b_7_*HADS-Anxiety + *b_8_*HADS-Depression + *b_9_*MSNQ + *ε*
2. CLS Discrepancy = *b_0_* + *b_1_*age + *b_2_*MS duration + *b_3_*time since last relapse + *b_4_*presence of a neurodevelopmental condition + *b_5_*MSIS-29 + *b_6_*MFIS total + *b_7_*HADS-Anxiety + *b_8_*HADS-Depression + *b_9_*MSNQ + *ε*
3. CRI-Work = *b_0_* + *b_1_*age + *b_2_*MS duration + *b_3_*time since last relapse + *b_4_*presence of a neurodevelopmental condition + *b_5_*MSIS-29 + *b_6_*MFIS total + *b_7_*HADS-Anxiety + *b_8_*HADS-Depression + *b_9_*MSNQ + *ε*
4. CRI-Leisure = *b_0_* + *b_1_*age + *b_2_*MS duration + *b_3_*time since last relapse + *b_4_*presence of a neurodevelopmental condition + *b_5_*MSIS-29 + *b_6_*MFIS total + *b_7_*HADS-Anxiety + *b_8_*HADS-Depression + *b_9_*MSNQ + *ε*

CLS Discrepancy scores were calculated by subtracting CLS Recent scores from CLS 20s scores. Regression diagnostics and sensitivity analyses were conducted. Values were considered potential (1) regression outliers if their studentised residuals > |2|, (2) high leverage cases if their hat values > 2*Mean_hat_ and (3) high influence cases if Cook’s distance *D_i_* > 4 ÷ (*n* − *k* − 1). Data were excluded if removing potential high influence cases improved model fit.

The significance level for all analyses is alpha 0.001 (<0.01 interpreted as a trend). This alpha level was set to guard against type 1 error in the context of multiple comparisons and correlations. Exact *p*-values were reported throughout to allow readers to interpret significance individually.

Results were presented to a Public and Patient Involvement (PPI) panel of pwMS (*n* = 20) and their insights contributed to the interpretation of results and implications in the discussion section.

## Results

Participants included 206 pwMS and 150 age- and gender-matched controls (Supplementary Materials 3 contains participant flow chart). PwMS had a mean age of 40.9 years (standard deviation (SD) = 9.9), disease duration of 8.7 years (SD = 7.2) and last reported relapse 2.8 years ago (SD = 3.4). Most (80.1%) pwMS reported having relapsing–remitting MS and 74.7% reported taking DMT (demographic data in Tables 1 and 2).

**Table 1.** Demographic data for pwMS and controls.

	MS	Controls
Age		
M (SD)	40.9 (9.9)	39.4 (10.2)
Range	20–60	26–60
Gender (%)		
Woman	78.6% (*n* = 162)	72.0% (*n* = 108)
Man	19.9% (*n* = 41)	27.3% (*n* = 41)
Non-Binary Person	1.0% (*n* = 2)	-
Self-Identification^a^	0.5% (*n* = 1)	0.7% (*n* = 1)
MS subtype		
RRMS	80.1% (*n* = 165)	-
SPMS	7.3% (*n* = 15)	-
PPMS	6.8% (*n* = 14)	-
Not sure	5.8% (*n* = 12)	-
Ethnicity^b^ (%)		
Arab	-	0.7% (*n* = 1)
Asian – Chinese	1.0% (*n* = 2)	1.3% (*n* = 2)
Asian – Indian/Pakistani/Bangladeshi	1.0% (*n* = 2)	2.0% (*n* = 3)
Asian – any other Asian background	-	0.7% (*n* = 1)
Black – African	7.3% (*n* = 15)	9.3% (*n* = 14)
Black – any other Black background	0.5% (*n* = 1)	-
Other including mixed background	1.0% (*n* = 2)	4.7% (*n* = 8)
White	88.8% (*n* = 183)	80.7% (*n* = 121)
White Roma	0.5% (*n* = 1)	-
White Traveller	-	0.7% (*n* = 1)
Work status (%)		
Employed	68.3% (*n* = 140)	60.0% (*n* = 90)
Unemployed	12.2% (*n* = 23)	13.3% (*n* = 20)
Retired	4.9% (*n* = 10)	4.0% (*n* = 6)
Not working due to disability	4.4% (*n* = 9)	-
Long-term sick leave	1.0% (*n* = 2)	-
Self-employed	1.0% (*n* = 2)	4.0% (*n* = 6)
Unpaid care work	4.9% (*n* = 10)	1.3% (*n* = 2)
Student	1.5% (*n* = 3)	14.7% (*n* = 22)
Student and employed	2.9% (*n* = 6)	2.7% (*n* = 4)
Missing	1/206	-
Additional neurodevelopmental/neurological condition (%)		
Yes	18.5% (*n* = 38)	6.7% (*n* = 10)
No	81.6% (*n* = 168)	93.3% (*n* = 140)

a Participants in this category chose to self-identify their gender, rather than selecting one of the three categories (Woman, Man, Non-Binary Person).

b Ethnicity categories were adapted from the 2022 Irish Census categories.

RRMS = relapsing–remitting MS; SPMS = secondary progressive MS; PPMS = primary progressive MS.

See Supplementary Materials 3 for further information on additional neurodevelopmental/neurological conditions.

**Table 2.** Prevalence of common MS symptoms self-reported by pwMS.

	Yes	No	Not sure
Fatigue			
Pre-MS	46.1% (*n* = 95)	46.6% (*n* = 96)	7.3% (*n* = 15)
Post-MS	88.8% (*n* = 183)	9.2% (*n* = 19)	1.9% (*n* = 4)
Depression			
Pre-MS	53.9% (*n* = 111)	37.4% (*n* = 77)	8.7% (*n* = 18)
Post-MS	81.1% (*n* = 167)	15.1% (*n* = 31)	3.9% (*n* = 8)
Anxiety			
Pre-MS	49.5% (*n* = 102)	45.6% (*n* = 94)	4.9% (*n* = 10)
Post-MS	75.2% (*n* = 155)	19.9% (*n* = 41)	4.9% (*n* = 10)

Most pwMS (65.5%) and controls (90.0%) were recruited through social media. There was no significant main effect of recruitment stream (CRI (*p* = 0.022–0.822), CLS (*p* = 0.032–0.362), MFIS (*p* = 0.238–0.945), HADS (*p* = 0.173–0.225), MSNQ (*p* = 0.012)). Thus, our method of recruitment was deemed robust.

### CR statistics

PwMS and controls had relatively high CRI scores (Tables 3 and 4). PwMS reported high levels of MS-impact and fatigue, with a large proportion exceeding the clinical cut-off for depression (43.7%), anxiety (59.2%) and cognitive difficulty (52.9%) (Tables 5 and 6).

**Table 3.** Descriptive statistics of CR measures for pwMS and controls.

	MS		Controls		Group comparison^a^		
	M (SD)	Range	M (SD)	Range	*W*	*p*	Effect size^b^
CLS							
20s	17.4 (4.9)	7–30	17.2 (4.3)	8–26	15,852	0.675	0.03
Recent	14.4 (4.7)	7–30	16.7 (4.5)	7–29	11,072	**<0.001**	-0.28
CRI							
Education	110.1 (11.7)	71–145	112.5 (10.7)	77–140	12,806	0.006	-0.17
Work	106.6 (11.2)	84–151	102.2 (8.8)	85–137	19,206	**<0.001**	0.24
Leisure	107.6 (17.2)	77–167	107.8 (17.3)	78–165	15,431	0.984	-0.00
Total	110.4 (13.1)	85–159	109.6 (11.4)	86–141	15,896	0.642	0.03

CLS = Cognitive Leisure Scale (raw scores can range from 7 to 35); CRI = Cognitive Reserve Index questionnaire (low (< 70), medium-low (70–84), medium (85–114), medium-high (115–130), high (>130)); significant differences are highlighted in bold.

a Group comparisons conducted using Mann–Whitney *U* tests.

b Effect size is given by rank biserial correlation (values between 0 and|0.29| interpreted as small effect size, between|0.30| and|0.49| as moderate effect size and between|0.50| and|1| as large effect size).

**Table 4.** Frequency of pwMS and controls in each of total CRI groups.

	MS	Controls
Low (<70)	0%	0%
Medium-low (70–84)	0%	0%
Medium (85–114)	69.9% (*n* = 144)	67.3% (*n* = 101)
Medium-high (115–130)	22.3% (*n* = 46)	26.7% (*n* = 40)
High (>130)	7.8% (*n* = 16)	6.0% (*n* = 9)

CRI = Cognitive Reserve Index questionnaire.

**Table 5.** Descriptive statistics of self-report measures for pwMS and controls.

	MS		Controls		Group comparison^a^		
	M (SD)	Range	M (SD)	Range	*W*	*p*	Effect size^b^
MSIS-29							
Total	72.2 (28.1)	29–142	-	-	-	-	-
MFIS							
Physical	18.4 (10.2)	0–36	10.7 (7.8)	0–35	22,309	**<0.001**	0.44
Cognitive	19.7 (10.4)	0–40	13.8 (8.9)	0–39	20,665	**<0.001**	0.34
Psychosocial	4.0 (2.5)	0–8	2.6 (2.1)	0–8	20,432	**<0.001**	0.32
Total	42.1 (21.5)	0–84	27.1 (17.4)	0–77	21,873	**<0.001**	0.42
HADS							
Anxiety	9.2 (5.0)	0–21	7.5 (4.5)	0–19	18,502	0.001	0.20
Depression	6.9 (4.3)	0–20	4.4 (3.8)	0–19	20,802	**<0.001**	0.35
MSNQ							
Total	27.5 (14.1)	0–58	18.9 (11.5)	1–56	21,123	**<0.001**	0.37

MSIS-29 = MS Impact Scale (raw scores can range from 29 to 145); MFIS = Modified Fatigue Impact Scale (total raw scores can range from 0 to 84, with scores >38 indicating high levels of fatigue); HADS = Hospital Anxiety and Depression Scale (raw scores can range from 0 to 21 on each subscale, with scores >7 indicating presence of anxiety/depression); MSNQ = MS Neuropsychological Questionnaire (raw scores can range from 0 to 60, with scores >27 indicating presence of cognitive difficulty).

Significant differences are highlighted in bold.

a Group comparisons conducted using Mann–Whitney *U* tests.

b Effect size is given by rank biserial correlation (values between 0 and|0.29| interpreted as small effect size, between|0.30| and|0.49| as moderate effect size and between|0.50| and|1| as large effect size).

**Table 6.** Frequency of pwMS and controls with clinical levels of common MS symptoms.

	MS	Controls
Fatigue (MFIS)		
Low (total ⩽ 38)	40.8% (*n* = 84)	74.7% (*n* = 112)
High (total > 38)	59.2% (*n* = 122)	25.3% (*n* = 38)
Depression (HADS-D)		
Subclinical (0–7)	56.3% (*n* = 116)	80.0% (*n* = 120)
Mild (8–10)	25.2% (*n* = 52)	12.7% (*n* = 19)
Moderate (11–14)	11.7% (*n* = 24)	5.3% (*n* = 8)
Severe (15–21)	6.8% (*n* = 14)	2.0% (*n* = 3)
Anxiety (HADS-A)		
Subclinical (0–7)	40.8% (*n* = 84)	53.3% (*n* = 80)
Mild (8–10)	19.4% (*n* = 40)	21.3% (*n* = 32)
Moderate (11–14)	22.8% (*n* = 47)	17.3% (*n* = 26)
Severe (15–21)	17.0% (*n* = 35)	8.0% (*n* = 12)
Cog Difficulty (MSNQ)		
Low (total ⩽ 27)	47.1% (*n* = 97)	78.0% (*n* = 117)
High (total > 27)	52.9% (*n* = 109)	22.0% (*n* = 33)

MFIS = Modified Fatigue Impact Scale (total raw scores can range from 0 to 84); HADS = Hospital Anxiety and Depression Scale (raw scores can range from 0 to 21 on each subscale); MSNQ = MS Neuropsychological Questionnaire (raw scores can range from 0 to 60).

### Recent CR-building and neuropsychological outcomes in pwMS

Recent engagement in cognitively enriching leisure activities (CLS Recent) was negatively associated (weak-moderate) with self-reported cognitive difficulties (MSNQ) (rho = −0.31, *p* < 0.001). Thus, greater engagement in cognitively enriching activities was associated with less cognitive difficulty.

Recent engagement in cognitively enriching leisure activities was also negatively associated (weak-moderate) with cognitive fatigue (MFIS cognitive; rho = −0.27, *p* < 0.001) and total fatigue (MFIS total; rho = −0.24, *p* < 0.001), and depression (HADS-D; rho = −0.31, *p* < 0.001). There was a trend towards significance with MS-impact (MSIS-29; rho = −0.19, *p* = 0.005), physical fatigue (MFIS physical; rho = −0.19, *p* = 0.006) and anxiety (HADS-A; rho = −0.21, *p* = 0.003).

Controlling for fatigue (cognitive and total) and depression in a partial correlation, the association between recent leisure and cognitive difficulty was not significant (*p* = 0.017).

### Differences in CR across pwMS and controls

#### Early 20s and recent engagement in cognitively enriching leisure activities

**Figure 1.**
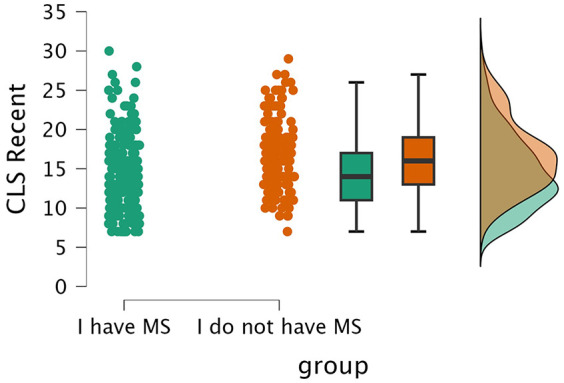
Recent engagement in cognitively enriching leisure activities (CLS Recent) across pwMS and controls.

Consistent with our hypotheses, pwMS and controls did not differ in engagement in cognitively enriching leisure activities reported during their early 20s (CLS 20s; *W* = 15,852, *p* = 0.675). However, they differed in recent engagement (CLS Recent; *W* = 11,072, *p* < 0.001, effect size (rank biserial correlation) = −0.28), with controls (median = 16) reporting greater engagement in cognitively enriching leisure activities than pwMS (median = 14) (Figure 1).

**Figure 2.**
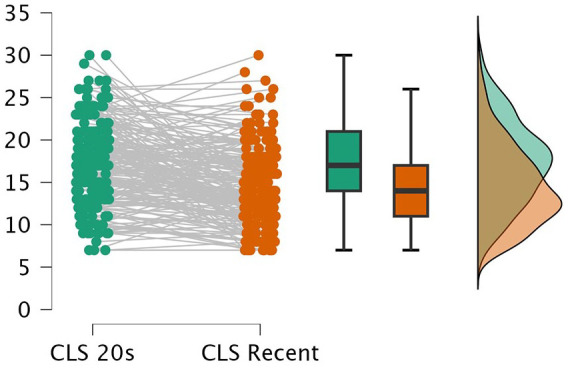
PwMS’ engagement in cognitively enriching leisure activities during their early 20s (CLS 20s) versus last year (CLS Recent).

Controls’ recent level of engagement in cognitively enriching leisure activities did not differ from their early 20s (*V* = 4724.5, *p* = 0.052), while pwMS reported higher engagement (*V* = 13,948, *p* < 0.001, effect size (rank biserial correlation) = 0.60) in their early 20s (median = 17) compared to recent engagement (median = 14) (Figure 2).

#### CR developed throughout the lifespan

There was a trend towards pwMS having lower CRI-Education scores than controls (*W* = 12,806, *p* = 0.006, Median_PwMS_ = 109, Median_Controls_ = 113). PwMS had higher CRI-Work scores than controls (*W* = 19,206, *p* < 0.001, Median_PwMS_ = 105, Median_Controls_ = 101, effect size (rank biserial correlation) = 0.24). A Kruskal–Wallis test revealed a difference in CRI-Work even when controlling for education (*X*^2^(1, 356) = 15.37, *p* < 0.001, effect size [*ε*^2^] = 0.04). There was no evidence groups differing in CRI-Leisure (*W* = 15,430, *p* = 0.984) or total CRI (*W* = 15,896, *p* = 0.642). CRI scores were not correlated with any self-report measure.

### The relationship of common MS symptoms with CR

The multiple regression model predicting recent engagement in cognitively enriching leisure activities (CLS Recent, Table 7) revealed that higher levels of depression (HADS-D) and self-reported cognitive difficulties (MSNQ) were associated with lower engagement.

**Table 7.** Summary of the multiple regression model predicting CLS Recent.

		95% CI					
	*B*	LL	UL	*SE*	*β*	*t*	*p*
(Intercept)	17.43	14.36	20.50	1.56	–	11.21	<0.001
Age	0.00	-0.06	0.07	0.03	0.01	0.09	0.930
MS duration	-0.04	-0.13	0.048	0.04	-0.06	-0.91	0.366
Time since last relapse	0.01	-0.21	0.18	0.10	-0.04	-0.13	0.893
Neuro condition: Yes	0.22	-1.36	1.81	0.80	0.05	0.28	0.781
MSIS-29	0.01	-0.03	0.05	0.02	0.04	0.68	0.495
MFIS total	0.03	-0.03	0.09	0.03	0.05	0.86	0.392
HADS-Anxiety	0.08	-0.08	0.24	0.08	0.06	0.97	0.335
**HADS-Depression**	**-0.41**	**-0.61**	**-0.22**	**0.10**	**-0.30**	**-4.22**	**<0.001**
**MSNQ**	**-0.11**	**-0.17**	**-0.05**	**0.03**	**-0.27**	**-3.56**	**<0.001**

Eleven influential cases were removed (see Supplementary Materials 4 for original model).

Adjusted *R*^2^ = 16%; *F*(9, 185) = 5.16, *p* < 0.001.

CI = confidence interval; LL = lower limit; UL = upper limit; MSIS-29 = MS Impact Scale; MFIS = Modified Fatigue Impact Scale; HADS = Hospital Anxiety and Depression Scale; MSNQ = MS Neuropsychological Questionnaire.

Significant predictors are highlighted in bold.

Higher levels of depression (HADS-D) were also associated with a higher discrepancy (i.e. lower CLS Recent compared to CLS 20s scores, resulting in positive and higher discrepancy scores) between recent engagement in cognitively enriching leisure activities and engagement in the early 20s (Table 8).

**Table 8.** Summary of the multiple regression model predicting CLS 20s – CLS Recent discrepancy scores.

		95% CI					
	*B*	LL	UL	*SE*	*β*	*t*	*p*
(Intercept)	-3.68	-6.78	-0.59	1.57	–	-2.35	0.020
Age	0.02	-0.04	0.09	0.03	0.05	0.69	0.488
MS duration	0.11	0.03	0.20	0.05	0.18	2.53	0.012
Time since last relapse	-0.06	-0.24	0.13	0.09	-0.04	-0.60	0.549
Neuro condition: Yes	0.37	-1.16	1.91	0.78	0.03	0.48	0.633
MSIS-29	-0.00	-0.04	0.04	0.02	-0.01	-0.11	0.917
MFIS total	0.07	0.01	0.12	0.03	0.30	2.16	0.032
HADS-Anxiety	-0.15	-0.32	0.02	0.09	-0.16	-1.75	0.083
**HADS-Depression**	**0.39**	**0.19**	**0.60**	**0.10**	**0.35**	**3.84**	**<** **0.001**
MSNQ	0.03	-0.03	0.08	0.03	0.07	0.90	0.372

Eleven influential cases were removed (see Supplementary Materials 4 for original model).

Adjusted *R*^2^ = 29%; *F*(9, 185) = 9.86, *p* < 0.001.

CI = confidence interval; LL = lower limit; UL = upper limit; MSIS-29 = MS Impact Scale; MFIS = Modified Fatigue Impact Scale; HADS = Hospital Anxiety and Depression Scale; MSNQ = MS Neuropsychological Questionnaire.

Significant predictors are highlighted in bold.

In contrast, greater occupational achievement (CRI-Work, Table 9) and leisure activity across the adult lifespan (CRI-Leisure, Table 10) were only associated with older age.

**Table 9.** Summary of the multiple regression model predicting CRI-Work.

		95% CI					
	*B*	LL	UL	*SE*	*β*	*t*	*p*
(Intercept)	84.78	78.91	90.65	2.98	–	28.49	<0.001
**Age**	**0.62**	**0.50**	**0.75**	**0.07**	**0.66**	**9.57**	**<0.001**
MS duration	-0.15	-0.34	0.03	0.10	-0.11	-1.61	0.109
Time since last relapse	0.03	-0.35	0.40	0.19	0.01	0.13	0.893
Neuro condition: Yes	0.39	-2.50	3.28	1.47	0.02	0.27	0.789
MSIS-29	-0.06	-0.14	0.02	0.04	-0.19	-1.57	0.119
MFIS total	-0.08	-0.20	0.05	0.06	-0.17	-1.24	0.218
HADS-Anxiety	0.23	-0.09	0.56	0.16	0.13	1.41	0.159
HADS-Depression	-0.06	-0.44	0.32	0.19	-0.03	-0.30	0.765
MSNQ	0.10	-0.00	0.21	0.05	0.16	1.94	0.054

14 influential cases were removed (see Supplementary Materials 4 for original model); adjusted *R*^2^ = 36%; *F*(9, 182) = 13.01, *p* < 0.001.

CI = confidence interval; LL = lower limit; UL = upper limit; MSIS-29 = MS Impact Scale; MFIS = Modified Fatigue Impact Scale; HADS = Hospital Anxiety and Depression Scale; MSNQ = MS Neuropsychological Questionnaire.

Significant predictors are highlighted in bold.

**Table 10.** Summary of the multiple regression model predicting CRI-Leisure.

	*B*	95% CI		*SE*	*β*	*t*	*p*
		LL	UL				
(Intercept)	70.05	61.61	78.50	4.28	–	16.36	<0.001
**Age**	**1.06**	**0.88**	**1.25**	**0.09**	**0.71**	**11.43**	**<0.001**
MS duration	-0.24	-0.50	0.01	0.13	-0.12	-1.90	0.059
Time since last relapse	-0.58	-1.11	-0.05	0.27	-0.13	-2.15	0.033
Neuro condition: Yes	-1.09	-5.37	3.19	2.17	-0.03	-0.50	0.616
MSIS-29	-0.02	-0.13	0.09	0.06	-0.04	-0.39	0.699
MFIS total	0.13	-0.04	0.30	0.09	0.18	1.46	0.146
HADS-Anxiety	0.16	-0.29	0.62	0.23	0.06	0.71	0.476
HADS-Depression	-0.37	-0.93	0.18	0.28	-0.11	-1.33	0.184
MSNQ	-0.19	-0.36	-0.02	0.09	-0.18	-2.23	0.027

12 influential cases were removed (see Supplementary Materials 4 for original model).

Adjusted *R*^2^ = 44%.

*F*(9, 184) = 18.09, *p* < 0.001.

CI = confidence interval; LL = lower limit; UL = upper limit; MSIS-29 = MS Impact Scale; MFIS = Modified Fatigue Impact Scale; HADS = Hospital Anxiety and Depression Scale; MSNQ = MS Neuropsychological Questionnaire.

Significant predictors are highlighted in bold.

## Discussion

This study aimed to achieve a better understanding of CR-building in MS by investigating how engagement in enriching activities is associated with common invisible MS symptoms. We found that higher levels of recent engagement in cognitively enriching leisure activities were associated with lower levels of self-reported cognitive difficulty in pwMS, but only when fatigue and depression were not controlled for in the analysis. This raises the possibility that previously reported associations between CR and cognitive outcomes may be attributed, at least in part, to these factors.

PwMS and controls did not differ in reported engagement in cognitively enriching leisure activities during their early 20s. However, pwMS reported lower levels of recent engagement in cognitively enriching leisure activities compared to controls during the last year. PwMS also reported a decrease in engagement during the last year compared to their early 20s, while controls experienced no change between the two timepoints. These results align with our hypotheses and are consistent with prior research reporting that pwMS experienced greater decreases in leisure activities than controls, and that an association between leisure and cognitive outcomes was not significant when controlling for depression.^12^ Correspondingly, our regression models revealed that higher levels of depression were associated with lower and decreased engagement in cognitive leisure activities in pwMS.

In contrast, greater occupational achievement (CRI-Work) and engagement in a broader set of leisure activities undertaken across the entire adult lifespan (CRI-Leisure) were only associated with older age. The CRIq was developed for an older adult population. This could explain age as the only predictor in the context of our comparatively young sample. In addition, self-report measures, such as the ones used to assess participants’ experience of common MS symptoms, may be less related to lifetime enrichment as they only assess symptoms experienced during the last few weeks.

Previous research on CR and MS symptoms tended to investigate CR as a predictor, not an outcome. For example, a recent study investigated the moderating role of CR in the relationship between fatigue and depression.^23^ In a similar study, CR was estimated only through pre-MS enrichment.^24^ Thus, this study makes a unique contribution by focussing on CR-building throughout the lifespan (including post-diagnosis enrichment) and the role of MS symptoms in pwMS’ ability to engage in enriching activities.

### Limitations

Further factors and symptoms may have contributed to pwMS’ engagement in enriching activities. Notably, given the survey-based nature of this research, we did not collect data on participants’ disability level, for example using the Expanded Disability Status Scale.^25^ While the MSIS-29 captures the self-reported impact of physical and psychological symptoms on everyday life, future research should aim to more exhaustively assess and investigate possible MS-impact. For example, future research could use a self-report measure of disability, such as the Patient-Determined Disease Steps (PDDS).^26^ Another limitation of this research is that we administered the CRIq via a survey, instead of the traditional semi-structured interview. We could not verify MS diagnoses and relied on self-report. It is unlikely that participants accidentally self-reported living with MS as they were asked detailed questions about MS (e.g. MS duration, time since last relapse, DMT). Yet, this limitation remains. Similarly, there is a risk of self-report bias in using self-report measures of common MS symptoms and CR, albeit all measures used in this study have been validated.

### Implications and conclusion

Leisure activity may be the most modifiable aspect of CR post-diagnosis. Our findings highlight the importance of considering and addressing depression and fatigue, particularly in the context of lifestyle recommendations in clinical practice. Our PPI panel expressed that they would appreciate clinicians providing guidance around enriching activities and support in the ability to engage with these activities. Depression and fatigue are modifiable through pharmacological and non-pharmacological interventions.^27,28^ Around two thirds of our PPI panel also highlighted that they purposefully engage in cognitive leisure activities now, that they would not have pursued prior to their diagnosis. Some of these activities may not be well captured in existing measures assessing engagement in more traditional activities. In supporting pwMS in engaging in enriching leisure activities, through managing depression and fatigue, and building their CR, we may be able to better support pwMS in achieving better cognitive outcomes and quality of life.

## Supplemental Material

sj-docx-1-msj-10.1177_13524585251338757 – Supplemental material for Cognitive reserve in multiple sclerosis: The role of depression and fatigue Supplemental material, sj-docx-1-msj-10.1177_13524585251338757 for Cognitive reserve in multiple sclerosis: The role of depression and fatigue by Clara Stein, Fiadhnait O’Keeffe, Méadhbh Brosnan, Claire Flynn, Christopher McGuigan and Jessica Bramham in Multiple Sclerosis Journal
