# Supplementary material for: Cognitive reserve in multiple sclerosis: The role of depression and fatigue
Source: Mult Scler. 2025 Jun 11;31(8):995–1006. doi: 10.1177/13524585251338757 (PMC12228891; doi:10.1177/13524585251338757)
Supplement: sj-docx-1-msj-10.1177_13524585251338757 – Supplemental material for Cognitive reserve in multiple sclerosis: The role of depression and fatigue [file sj-docx-1-msj-10.1177_13524585251338757.docx]

**Supplementary Materials 1**

**Rationale for CRI computing decisions**

Traditionally, the CRIq is administered as a semi-structured interview. We aimed to approximate this structure with our survey. Thus, participants used free text fields to report years spent in formal education and vocational training (CRI-Education), the titles and durations of all paid jobs lasting at least a year (CRI-Work), and the number of years for which leisure activities were carried out often/always (CRI-Leisure). Given the nature of free text fields, some responses were not provided in the desired format. For consistency and transparency, we created an overview of decisions made whilst computing CRI scores.

The following rationale outlines some of the thinking behind the decision made during the CRI computing process. Our overall goal was to adopt a consistent and transparent approach and to avoid over- or underestimation of engagement in enriching activities. More examples and greater detail can be found in Table S1.

CRI-Education:

If no exact years of education were provided, but a degree was mentioned (e.g., "Graduated with a Bachelor of Science plus some Graduate studies"), we awarded the typical years spent in primary and secondary school in Ireland (14 years) plus the typical duration of a Bachelor’s degree (3 years), resulting in a total of 17 years of education. To avoid overestimation, the lower end of typical degree duration was used and we decided that “some Graduate studies” would be too vague and short to assume that this person completed a full year of graduate studies.

CRI-Work:

We used common sense to make meaning of commonly used job abbreviations. However, if participants only stated the number of years for which they were employed, or only their job titles, but no duration, we decided to treat this as missing data as we cannot assume which jobs or for how long jobs were carried out.

CRI-Leisure:

Some participants provided responses which exceeded the participant’s adult life, but were otherwise plausible. For example, one participant stated that they had been reading books frequently for 40 years, despite only being aged 50. We assumed this participant forgot they were supposed to only consider their adult life and imputed the highest possible value of 32 years (50-18).

**Table S1.** Overview of CRI computation decisions

| Item | Issue | Example | Decision | Reasoning |
| --- | --- | --- | --- | --- |
| CRI-Edu | Only college level education is mentioned, but no primary or secondary education | "6 years of college education" | Average years in primary+secondary education added (14 years) | For consistency, Irish standard years (13 or 14) will be used - a leaving cert or equivalent qualification is typically required for college-level education (alternative routes exist but are less common) |
|  | No exact years for any level of education provided, but a degree or college is mentioned | "Graduated with a Bachelor of Science plus some Graduate studies" | Above + average time for the degree type added (Bachelor = 3 years; Master's = 1; PhD = 3 years) - in this case, we will be conservative and use the lower end of typical years per degree and also only count completed degrees | "some graduate studies" is too vague to assume this person completed a full year of graduate studies; lower end of typical years will be used so as to not overestimate someone's level of education |
|  |  | "Degree" | Lowest degree level (bachelor) will be assumed = 14+3 years | Avoid over or under estimation |
|  |  | "Leaving cert, PLC course, Diploma in Massage Therapy, Diploma in Sports Massage Therapy, Medical Secretary Diploma" | Leaving cert = 14, PLC = 1 year, diplomas not counted | Diplomas can vary in length and structure, typically, diplomas are done over the course of a few weeks-months max and they are typically less than the regular FT or PT education (often a block of classes or weekend classes etc.) |
|  |  | "higher certificate" | Add 3 years to primary+secondary | Typical duration |
|  |  | "some colleage" | Only average primary+secondary education will be imputed | Too vague to assume significant time was spent in higher level education |
|  | Range of years provided | "15-20" | Rounded average (17.5 à 18) will be imputed | Avoid over or under estimation |
|  | Part time | "5 years part time" | Counted as 2.5 years (3 years rounded) full time education | Most people provide years of full time education and years of education is used as indicator of educational level, so full time years are more representative |
|  | Years of education don't match job description -- too low for job | "6" for an engineer | Average years in primary+secondary education added (14 years) | Person most likely only considered their postgraduate education |
|  | Years of education don't match job description -- too high | Years of education and years of vocational training are the same and it is very unlikely that person did total number of years given their job | Disregard years of vocational training | Interpreted as person having misunderstood the question, they likely just expressed the same figure twice |
| CRI-Work | Job abbreviated | "it spec" | Job title will be deducted if abbreviation is common or reasonable enough, here: IT specialist | If people use common or reasonable abbreviations, then it is fair to assume that that's the job that was meant |
|  |  | "sna" | Special needs assistant |  |
|  | Only number of years mentioned | "9" | Missing data | We can't assume what type of job this is |
|  | Only type of job mentioned | "Kitchen Porter, Team member, Concierge" | Missing data | We can't assume how long these jobs lasted |
|  | Participant worked across more than 3 levels | level 1, 2, 3, 4 | Disregard lowest level job | CRIq computation excel only allows for 3 job levels |
| CRI-Leisure | Years exceed the person's adult life but are plausible (within lifespan) | Participant is aged 50 and states that they have been reading books often for 40 years | Highest possible value will be imputed (50-18=32) | Most likely an honest mistake, people often forget to only consider their adult life when responding |
|  | Years exceed the person's entire lifespan and are thus not plausible | Participant is aged 50 and states that they have been reading books often for 55 years | Missing data | Impossible value |
|  | Life period mentioned | "Early 20s and younger" | 5 years | Best estimate; avoid under or overestimation |
|  |  | "In 20s and early 30s" | 15 years |  |
|  | No years mentioned, but a frequency | "everyday", "daily" | Highest possible value will be imputed | Likely an activity that people have been doing very frequently their entire life |
|  |  | "3 times a week" | Highest possible value will be imputed | Likely an activity that people have been doing frequently their entire life |
|  | No years mentioned, but activities listed | "doctors appointment , weekend trips or outing" for driving | Never/rarely option will be selected | Person most likely doesn't drive 3 times a week or more |
|  |  | "WFH and social media" for new technologies | Highest possible value will be imputed | Most people have used new technologies all their life if middle aged |
|  |  | "Travelling once a quarter"; "book binges every couple of weeks" | Highest possible value will be imputed | Person seems to be doing annual activities clearly above the required threshold and this seems to be their norm |
|  |  | "Member of local rugby club" for social activities | Never/rarely option will be selected | Being a passive member of a club would not be sufficient to count as Often/Always -- avoid overestimation |
|  | Less than 1 year | "1 week" | Never/rarely option will be selected | Doing an activity for less than one year is not enough to select often/always - this will be rounded up to 5 years in the computation and 1 week of doing an activity does not justify this |
|  |  | "6 months" | Never/rarely option will be selected | Same as above |
|  | Often/always selected, but text response indicates it should have been never/rarely | "two times a week" | Never/rarely option will be selected | Most likely an honest mistake |

**Supplementary Materials 2**

**Table S2a.** Frequency of missing data

|  | MS | Controls |
| --- | --- | --- |
| Age | 0.49% (1/206) | 0% (0/150) |
| Ethnicity | 0% (0/206) | 0% (0/150) |
| Gender | 0% (0/206) | 0% (0/150) |
| Work status | 0.49% (1/206) | 0% (0/150) |
| Neurological/neurodevelopmental condition | 0% (0/206) | 0% (0/150) |
| MS duration | 0.49% (1/206) | - |
| Relapse | 19.90% (41/206) | - |
| CRI Education | 1.46% (3/206) | 2.67% (4/150) |
| CRI Work | 8.74% (18/206) | 2.00% (3/150) |
| CRI Leisure | 2.43% (5/206) | 2.00% (3/150) |
| CRI Total | 10.68% (22/206) | 6.00% (9/150) |
| CLS 20s | 0% (0/206) | 0% (0/150) |
| CLS Recent | 0% (0/206) | 0% (0/150) |
| MSIS-29 Total | 0% (0/206) | 0% (0/150) |
| MFIS Physical | 0% (0/206) | 0% (0/150) |
| MFIS Cognitive | 0% (0/206) | 0% (0/150) |
| MFIS Psychosocial | 0% (0/206) | 0% (0/150) |
| MFIS Total | 0% (0/206) | 0% (0/150) |
| HADS Anxiety | 0% (0/206) | 0% (0/150) |
| HADS Depression | 0% (0/206) | 0% (0/150) |
| MSNQ | 0% (0/206) | 0% (0/150) |

Notes. CRI = Cognitive Reserve Index; CLS = Cognitive Leisure Scale; MSIS-29 = MS Impact Scale; MFIS = Modified Fatigue Impact Scale; HADS = Hospital Anxiety and Depression Scale; MSNQ = MS Neuropsychological Questionnaire

**Table S2b.** Descriptive statistics of demographics for pwMS and controls based on complete and imputed data

|  | Complete | Imputed |
| --- | --- | --- |
| Age (years) | M = 40.92, SD = 9.93 | M = 40.92, SD = 9.91 |
| MS duration (years) | M = 8.75, SD = 7.25 | M = 8.73, SD = 7.24 |
| Time since last relapse (years) | M = 2.96, SD = 3.67 | M = 2.87, SD = 3.45 |

**Table S2c.** Descriptive statistics of CR measures for pwMS and controls based on complete data

|  | MS | | | | | Controls | | | | |
| --- | --- | --- | --- | --- | --- | --- | --- | --- | --- | --- |
|  | n | M | SD | Min | Max | n | M | SD | Min | Max |
| CLS |  |  |  |  |  |  |  |  |  |  |
| 20s | 206 | 17.40 | 4.94 | 7 | 30 | 150 | 17.18 | 4.30 | 8 | 26 |
| Recent | 206 | 14.43 | 4.71 | 7 | 30 | 150 | 16.66 | 4.49 | 7 | 29 |
| CRIq |  |  |  |  |  |  |  |  |  |  |
| Education | 203 | 110.05 | 11.76 | 71 | 145 | 146 | 112.37 | 10.79 | 77 | 140 |
| Work | 188 | 106.55 | 11.51 | 84 | 151 | 147 | 102.17 | 8.85 | 85 | 137 |
| Leisure | 201 | 107.51 | 17.29 | 77 | 167 | 147 | 107.66 | 17.39 | 78 | 165 |
| Total | 184 | 110.66 | 13.71 | 85 | 159 | 141 | 109.58 | 11.55 | 86 | 141 |

Notes. CLS = Cognitive Leisure Scale (raw scores can range from 7-35); CRI = Cognitive Reserve Index questionnaire; see Table 3a in results for a comparison with the imputed data

**Table S2d.** Frequency of pwMS and controls in each of total CRI groups based on complete cases

|  | MS | Controls |
| --- | --- | --- |
| Low (<70) | 0% | 0% |
| Medium-low (70-84) | 0% | 0% |
| Medium (85-114) | 67.39% (n = 124) | 68.09% (n = 96) |
| Medium-high (115-130) | 23.91% (n = 44) | 25.53% (n = 36) |
| High (>130) | 8.70% (n = 16) | 6.38% (n = 9) |

Notes. See Table 3b in results for a comparison with the imputed data

**Supplementary Materials 3**

**Figure S3.** Participant flow chart

Submissions excluded (**n = 171**)

Did not complete survey (n = 119)

Participant provided conflicting information re MS status (n = 14)

Participant provided impossible/implausible data (n = 9)

Participant is aged over 60 (n = 6)

Participant completed survey twice (n = 18)

Participant completed survey three times (n = 2)

Participant completed survey four times (n = 3)

Submissions excluded (**n = 6**)

PwMS with HNPP, PTS, CMT (n = 1)

PwMS with autoimmune encephalitis (n = 1)

PwMS with brain tumour (n = 1)

Control with epilepsy (n = 1)

Control with genetic retinal disease (n = 1)

Control with functional neurological disorder (n = 1)

Submissions excluded (**n = 35**)

Youngest controls (n = 35)

Note. HNPP = hereditary neuropathy with liability to pressure palsy; PTS = Parsonage Turner Syndrome; CMT = Charcot-Marie-Tooth disease

We excluded participants with neurological conditions likely to impact on survey responses. However, we did not exclude pwMS with epilepsy, as this could be secondary to MS, and we did not exclude pwMS and controls with neurodevelopmental conditions. 38 pwMS and 10 controls reported living with a neurodevelopmental or other condition other than MS. See Table S3 for more information on self-reported conditions within the final sample. Note that presence of a self-reported condition was not a significant predictor in any of the regression models.

**Table S3.** Self-reported conditions within the final sample

|  | MS | Controls |
| --- | --- | --- |
| ADD | n = 1 | n = 1 |
| ADD and BPD | n = 1 | - |
| ADHD | n = 10 | n = 2 |
| ADHD and dyslexia | n = 1 | - |
| ADHD (with depression and anxiety) | n = 1 | - |
| Being assessed for ADHD | - | n = 1 |
| Autism | n = 3 | - |
| Depression, anxiety, fatigue | n = 1 | - |
| Dyslexia | n = 4 | - |
| Dyslexia and being assessed for ADHD | n = 1 | - |
| Dyslexia and ADHD | n = 1 | - |
| Dyslexia, dyscalculia, and autism | n = 1 | - |
| Dyspraxia and autism | - | n = 1 |
| Epilepsy or epileptic seizures | n = 9 | - |
| Migraine | n = 1 | n = 1 |
| Neuromyelitis optica and anti mog positive | n = 1 | - |
| Tourette syndrome | - | n = 1 |
| Unspecified | n = 2 | n = 3 |

**Supplementary Materials 4**

**Original regression models**

**Table S4a.** Summary of the original multiple regression model predicting CLS Recent

|  |  | 95% CI | |  |  |  |  |
| --- | --- | --- | --- | --- | --- | --- | --- |
|  | *B* | LL | UL | *SE* | $\beta$ | *t* | *p* |
| (Intercept) | 17.83 | 14.44 | 21.21 | 1.72 | – | 10.39 | < 0.001 |
| Age | 0.01 | -0.07 | 0.08 | 0.04 | 0.01 | 0.18 | 0.857 |
| MS duration | -0.04 | -0.14 | 0.06 | 0.05 | -0.06 | -0.78 | 0.436 |
| Time since last relapse | -0.05 | -0.26 | 0.15 | 0.10 | -0.04 | -0.49 | 0.622 |
| Neuro condition: Yes | 0.64 | -1.05 | 2.33 | 0.86 | 0.05 | 0.75 | 0.454 |
| MSIS-29 | 0.01 | -0.04 | 0.05 | 0.02 | 0.04 | 0.27 | 0.789 |
| MFIS total | 0.01 | -0.05 | 0.08 | 0.03 | 0.05 | 0.35 | 0.727 |
| HADS-Anxiety | 0.06 | -0.13 | 0.24 | 0.09 | 0.06 | 0.62 | 0.537 |
| HADS-Depression | -0.33 | -0.55 | -0.11 | 0.11 | -0.30 | -2.97 | 0.003 |
| MSNQ | -0.09 | -0.15 | -0.03 | 0.03 | -0.27 | -2.91 | 0.004 |

Note. For model with influential cases removed see Table 5 in Results; adjusted R^2^ = 9%; F(9, 196) = 3.34, p < 0.001; CI = confidence interval; LL = lower limit; UL = upper limit; MSIS-29 = MS Impact Scale; MFIS = Modified Fatigue Impact Scale; HADS = Hospital Anxiety and Depression Scale; MSNQ = MS Neuropsychological Questionnaire

**Table S4b.** Summary of the original multiple regression model predicting CLS 20s - CLS Recent discrepancy scores

|  |  | 95% CI | |  |  |  |  |
| --- | --- | --- | --- | --- | --- | --- | --- |
|  | *B* | LL | UL | *SE* | $\beta$ | *t* | *p* |
| (Intercept) | -5.38 | -8.86 | -1.91 | 1.76 | – | -3.06 | 0.003 |
| Age | 0.05 | -0.03 | 0.12 | 0.04 | 0.08 | 1.20 | 0.232 |
| MS duration | 0.06 | -0.04 | 0.16 | 0.05 | 0.08 | 1.11 | 0.267 |
| Time since last relapse | -0.01 | -0.22 | 0.20 | 0.11 | -0.01 | -0.09 | 0.931 |
| Neuro condition: Yes | -0.07 | -1.81 | 1.66 | 0.88 | -0.01 | -0.08 | 0.935 |
| MSIS-29 | 0.03 | -0.02 | 0.07 | 0.02 | 0.13 | 1.10 | 0.272 |
| MFIS total | 0.04 | -0.02 | 0.11 | 0.03 | 0.18 | 1.28 | 0.203 |
| HADS-Anxiety | -0.13 | -0.32 | 0.06 | 0.10 | -0.12 | -1.30 | 0.196 |
| HADS-Depression | 0.37 | 0.15 | 0.60 | 0.11 | 0.30 | 3.31 | 0.001 |
| MSNQ | 0.03 | -0.03 | 0.10 | 0.03 | 0.09 | 1.06 | 0.291 |

Note. For model with influential cases removed see Table 6 in Results; adjusted R^2^ = 27%; F(9, 196) = 9.27, p < 0.001; CI = confidence interval; LL = lower limit; UL = upper limit; MSIS-29 = MS Impact Scale; MFIS = Modified Fatigue Impact Scale; HADS = Hospital Anxiety and Depression Scale; MSNQ = MS Neuropsychological Questionnaire

**Table S4c.** Summary of the original multiple regression model predicting CRI-Work

|  |  | 95% CI | |  |  |  |  |
| --- | --- | --- | --- | --- | --- | --- | --- |
|  | *B* | LL | UL | *SE* | $\beta$ | *t* | *p* |
| (Intercept) | 81.11 | 74.16 | 88.07 | 3.53 | – | 23.01 | < 0.001 |
| Age | 0.74 | 0.59 | 0.89 | 0.08 | 0.66 | 9.75 | < 0.001 |
| MS duration | -0.22 | -0.43 | -0.02 | 0.10 | -0.15 | -2.18 | 0.031 |
| Time since last relapse | -0.06 | -0.48 | 0.36 | 0.21 | -0.02 | -0.30 | 0.765 |
| Neuro condition: Yes | 0.87 | -2.60 | 4.34 | 1.76 | 0.03 | 0.49 | 0.622 |
| MSIS-29 | -0.03 | -0.12 | 0.06 | 0.05 | -0.07 | -0.63 | 0.531 |
| MFIS total | -0.12 | -0.26 | 0.01 | 0.07 | -0.24 | -1.79 | 0.075 |
| HADS-Anxiety | 0.39 | 0.01 | 0.77 | 0.19 | 0.18 | 2.05 | 0.042 |
| HADS-Depression | -0.13 | -0.58 | 0.31 | 0.23 | -0.05 | -0.59 | 0.556 |
| MSNQ | 0.06 | -0.06 | 0.19 | 0.06 | 0.08 | 0.98 | 0.329 |

Note. For model with influential cases removed see Table 7 in Results; adjusted R^2^ = 32%; F(9, 196) = 11.64, p < 0.001; CI = confidence interval; LL = lower limit; UL = upper limit; MSIS-29 = MS Impact Scale; MFIS = Modified Fatigue Impact Scale; HADS = Hospital Anxiety and Depression Scale; MSNQ = MS Neuropsychological Questionnaire

**Table S4d.** Summary of the original multiple regression model predicting CRI-Leisure

|  |  | 95% CI | |  |  |  |  |
| --- | --- | --- | --- | --- | --- | --- | --- |
|  | *B* | LL | UL | *SE* | $\beta$ | *t* | *p* |
| (Intercept) | 70.59 | 60.00 | 81.17 | 5.37 | – | 13.16 | < 0.001 |
| Age | 1.05 | 0.82 | 1.28 | 0.12 | 0.61 | 9.12 | < 0.001 |
| MS duration | 0.01 | -0.30 | 0.31 | 0.16 | 0.00 | 0.03 | 0.974 |
| Time since last relapse | -0.58 | -1.22 | 0.06 | 0.32 | -0.12 | -1.80 | 0.074 |
| Neuro condition: Yes | -0.94 | -6.22 | 4.35 | 2.68 | -0.02 | -0.35 | 0.727 |
| MSIS-29 | -0.05 | -0.19 | 0.09 | 0.07 | -0.09 | -0.75 | 0.457 |
| MFIS total | 0.07 | -0.13 | 0.28 | 0.10 | 0.09 | 0.71 | 0.481 |
| HADS-Anxiety | 0.12 | -0.46 | 0.70 | 0.29 | 0.03 | 0.40 | 0.689 |
| HADS-Depression | -0.60 | -1.28 | 0.08 | 0.34 | -0.15 | -1.75 | 0.083 |
| MSNQ | -0.02 | -0.21 | 0.17 | 0.10 | -0.02 | -0.20 | 0.840 |

Note. For model with influential cases removed see Table 8 in Results; adjusted R^2^ = 33%; F(9, 196) = 12.28, p < 0.001; CI = confidence interval; LL = lower limit; UL = upper limit; MSIS-29 = MS Impact Scale; MFIS = Modified Fatigue Impact Scale; HADS = Hospital Anxiety and Depression Scale; MSNQ = MS Neuropsychological Questionnaire
